# Characterizing Lifetime and Daily Experiences of Weight Stigma among Sexual Minority Women with Overweight and Obesity: A Descriptive Study

**DOI:** 10.3390/ijerph17134892

**Published:** 2020-07-07

**Authors:** Emily Panza, KayLoni Olson, Carly M. Goldstein, Edward A. Selby, Jason Lillis

**Affiliations:** 1 Weight Control and Diabetes Research Center, The Miriam Hospital, Providence, RI 02903, USA; kayloni_olson@brown.edu (K.O.); carly_goldstein@brown.edu (C.M.G.); jlillis@lifespan.org (J.L.); 2 Warren Alpert School of Medicine, Brown University, Providence, RI 02903, USA; 3 Department of Psychology, Rutgers, the State University of New Jersey, New Brunswick, NJ 08901, USA; eas268@psych.rutgers.edu

**Keywords:** weight bias, stigma, sexual identity, health disparities, obesity

## Abstract

Sexual minority women are disproportionately impacted by obesity yet are underrepresented in weight stigma research. This Ecological Momentary Assessment (EMA) study is a secondary analysis that aimed to elucidate the frequency and contextual characteristics of perceived experiences of lifetime and momentary weight stigma among sexual minority women with overweight/obesity. Participants were 55 sexual minority women ages 18–60 with a body mass index ≥25 kg/m^2^. Perceived lifetime weight stigma events were assessed at baseline. For the subsequent five days, participants used a smartphone to complete five daily, random EMA prompts assessing the frequency/characteristics of perceived weight stigma events in daily life. All participants reported at least one lifetime weight stigma event. During the EMA period, participants reported 44 momentary weight stigma events (*M* = 0.80), with 24% of participants reporting at least one event. During most instances of weight stigma, women perceived the stigma’s cause to be their weight and another minority identity (e.g., sexual orientation). Findings showing high rates of perceived lifetime weight stigma in this sample and frequent co-occurrence of perceived weight stigma with stigma due to other marginalized identities in daily life underscore the need for future, larger studies investigating weight stigma through an intersectional lens in sexual minority women with overweight/obesity.

## 1. Introduction

Despite the high prevalence of obesity in the U.S. [1], weight-based stigma is one of the most pervasive and socially-accepted forms of bias [2,3]. This bias is fueled by negative stereotypes that are linked to obesity, such as being lazy or unattractive [4]. Weight stigma occurs when individuals experience unfair treatment based on body weight, such as discrimination or harassment [2]. Although societal standards promoting thinness contribute to a culture where weight stigma can impact individuals of any body weight, people with overweight/obesity experience weight stigma more frequently and intensely than their lower-weight counterparts [5]. Perceived weight stigma experiences have been linked to negative mental health outcomes like depression [6], reduced quality of life [7], and behaviors like binge eating and exercise avoidance [8,9], which are likely to impair both weight management and well-being [10]. Perceived weight discrimination also partially explains longitudinal associations between obesity and poor cardiometabolic health [11], suggesting that obesity-related health consequences are explained not only by the physical effects of greater body adiposity but also by the social stigma of obesity.

However, sexual orientation is rarely assessed or examined in weight stigma research. Obesity is prevalent among sexual minority women [12], yet little is known about weight stigma experiences in this group. Sexual minority women include women who self-identify with a sexual minority orientation (e.g., lesbian, bisexual) or who report same-sex attraction or behavior [13]. Many sexual minority women face discrimination due to sexual orientation (i.e., heterosexism) and gender, i.e., sexism [14], and 61–76% of sexual minority women also have overweight/obesity [15,16] and may experience additional stigma due to their body weight (i.e., weight stigma). It is imperative to investigate sexual minority women’s perceptions of weight stigma as this group is already at risk for other forms of unfair treatment [14].

Indeed, recent cross-sectional research suggests that weight stigma may be prevalent among sexual minority women. In a national sample of sexual minority youth of all weight categories, 50–55% of youth reported experiencing weight stigma from family and peers [17], mirroring rates seen in the general population of youth [18,19]. Among a national sample of adults seeking weight loss treatment [20], 69–79% of sexual minority adults reported experiencing weight stigma at some point in their lives, rates similar to those reported by heterosexual adults in the sample. Finally, in a study of young adult lesbian women of all weight statuses, 26% reported experiencing weight stigma once or more in the past month [21]. These studies indicate that subjective experiences of weight stigma may be common among sexual minority women, highlighting the need for more work in this area.

When assessing the frequency and characteristics of perceived weight stigma experiences, most of the weight stigma literature relies largely on one-time retrospective self-reports [22,23]. Among adults in the general population, these assessments show that weight stigma is often perpetrated by friends, family, and strangers, stigma events occur several times per year at various locations (e.g., at home, in public, at work), and weight stigma events often occur in the form of verbal comments [22,24]. Technological innovations have advanced our understanding of how weight stigma manifests in daily life by using Ecological Momentary Assessment (EMA) [25]. EMA allows participants to complete real-time assessments of their experiences as they go about their daily lives. This approach is advantageous because it enhances the ecological validity of data [26] and decreases problems with recall bias [27] due to the reduced time between an event and assessment of that event [26]. EMA has been applied to investigate the phenomenology of momentary weight stigma events among adults with overweight/obesity [28,29,30], but we are not aware of any EMA studies documenting daily weight stigma events among sexual minority women with overweight/obesity. Such work could elucidate the form and contexts in which sexual minority women endorse weight stigma. This novel work is needed to improve understanding of weight stigma in this understudied group.

### The Current Study

To fill this gap, we used data from a larger EMA study to assess the frequency and contextual characteristics of lifetime and momentary perceived weight stigma events in a sample of 55 sexual minority women with overweight/obesity. Participants self-reported subjective experiences of lifetime weight stigma and demographics at baseline, then subsequently completed a five-day EMA protocol involving multiple daily assessments of perceived weight stigma experiences. This descriptive study tested two aims. The first aim was to assess the frequency and characteristics of lifetime weight stigma in a sample of sexual minority women of higher body weight at baseline. The second aim was to use ecologically valid methods (i.e., EMA) to describe the frequency, characteristics (e.g., intensity), and contextual factors (e.g., location) of weight stigma experiences in daily life in this sample. Given the lack of prior research in this area and this study’s modest sample size, the goal of this study was primarily to provide descriptive pilot data to inform the design of future, larger studies investigating weight stigma events among sexual minority women.

## 2. Materials and Methods

This project was part of a larger study that was designed to assess the effects of multiple sources of stigma (e.g., heterosexism, sexism, weight stigma) on dysregulated eating behaviors among sexual minority women with overweight/obesity [31].

### 2.1. Participants

This study recruited 55 sexual minority women assigned female at birth with a BMI over 25 kg/m^2^. Sexual minority men were not recruited based on prior work showing that sexual minority men have a lower prevalence of overweight/obesity than their heterosexual counterparts [32,33], as well as research showing that women are disproportionately impacted by weight stigma [34]. Although sexual orientation is defined in myriad ways (e.g., sexual attraction, sexual behavior, self-identification), this study defined sexual minority status by self-identification with a sexual minority sexual orientation (e.g., gay/lesbian, bisexual, pansexual) to be consistent with most research in this area [12,13]. Participants were included in the study if they owned a smartphone and spoke English fluently. Participants were not eligible to participate if they reported current psychotic symptoms, past schizophrenia, or a developmental disorder, as these conditions may impair the ability to provide informed consent. Participants were not enrolled if they were pregnant, had a current serious medical condition (e.g., cancer), or had a history of weight loss surgery because these factors could impact experiences of hunger and satiety.

### 2.2. Procedures

All study procedures were approved by the Institutional Review Board at Rutgers, the State University of New Jersey (IRB#16-778M). Participant consent and IRB approval was obtained to publish aggregate rather than individual data due to the sensitive nature of the information collected in this multiply marginalized population. Participants were recruited from the community-at-large using online and in-person flyers advertising a “research study on stress and eating” for sexual minority women. Interested individuals completed an online screening survey. Potentially eligible women were provided with study information and were invited to attend a 65-min, in-person baseline visit. During this visit, women provided informed consent, completed baseline surveys using Qualtrics software (Qualtrics, Provo, UT, USA) [35], and height and weight were measured to compute BMI. Participants downloaded the study smartphone application hosted by LifeData (LifeData, LLC, Marion, IN, USA) [36] on their smartphone and received training in using the application (e.g., key terms like stigma were defined, concrete examples of stigma events were provided). Participants then completed a five-day EMA protocol (see EMA Measures for details). Participants received USD 15 for completing the baseline visit and USD 30 for completing the EMA period, regardless of their response rate.

### 2.3. Baseline Measures

#### 2.3.1. Demographics

Participants reported their racial and ethnic background, age, and education level. Biological sex and gender identity were assessed separately.

#### 2.3.2. Sexual Minority Status

Participants were asked, “Do you consider yourself to be” and chose from response options including “straight/heterosexual”, “gay/lesbian/homosexual”, “bisexual or pansexual”, “queer”, “asexual”, “unsure”, and “other”.

#### 2.3.3. Body Mass Index (BMI)

A calibrated digital scale and stadiometer were used to measure weight to the nearest 0.1 kg and height to the nearest millimeter to compute BMI (kg/m^2^). Participants were weighed in light clothing without shoes.

#### 2.3.4. Perceived Experiences of Lifetime Weight Stigma

Subjective perceptions of lifetime experiences of weight-based stigmatization were assessed using the Stigmatizing Situations Inventory (SSI) [22]. This 50-item measure asked participants to report the frequency of lifetime experiences of weight-based stigmatization across a range of settings on a scale from 0 (never) to 9 (daily). Sample items included, “Having people assume you have emotional problems because you are overweight” and “Being stared at in public.” The scale has been used extensively to measure stigmatization in populations with overweight/obesity [29,37]. This measure contains a global overall score and 11 sub-factors. We obtained good reliability for both the global factor (α = 0.93) and the subscales.

### 2.4. EMA Measures

For the five days following the baseline visit, participants used the app to complete five prompted assessments of perceived weight stigma per day. Prompts occurred randomly between 9:00 AM and 9:30 PM, and were at least two hours apart. Prompts not answered within 75 min of receipt counted as missing data, a standard that has been used in prior EMA studies [38].

#### Perceived Experiences of Momentary Weight Stigma

At every EMA prompt, participants reported whether they had experienced stigma since the prior EMA prompt. Specific items and response options are outlined in Table 1. Participants who endorsed stigma used seven items to provide details about each stigmatizing event they reported. For example, participants reported the perceived reason(s) for the stigma event, with the option to select multiple reasons from a checklist including weight, sexual orientation, gender, etc. Any stigma event attributed to weight was considered a weight stigma episode. Participants’ affective response to the stigma event, and the stigma intensity, location, source, and description were also assessed. The momentary assessment used in this study was adapted from existing measures of baseline and momentary stigma, including one prior study showing that adults can reliably report momentary weight stigma [3,22,23,30,39,40,41].

### 2.5. Data Analytic Strategy

Given the goal of the study to characterize perceived weight stigma events, along with the modest sample size and rate of daily weight stigma reported in this study, we primarily employed descriptive rather than inferential statistics. To determine the frequency of lifetime weight stigma within this sample, we computed the mean values for each subscale and global score of the SSI measure from the baseline assessment. Participants who endorsed experiencing weight stigma “once in your life” or more on any item of the SSI were coded as experiencing weight stigma on the overall measure and on that specific subscale. To determine the frequency and characteristics of perceived weight stigma in daily life, we analyzed descriptive data for all weight stigma events reported during EMA. We also sought to maximize statistical power by computing the overall frequency of perceived weight stigma events reported per participant during the EMA period.

We also conducted exploratory analyses to assess whether key variables like lifetime weight stigma, demographics (e.g., age, BMI), and compliance to EMA procedures were associated with the total number of weight stigma episodes reported during the EMA period. To do this, we used non-multilevel generalized linear models with a Poisson distribution and log link function to account for the non-linear (i.e., count) distribution of the weight stigma frequency variable. Each variable was modeled separately to preserve statistical power, though we also modeled SSI score simultaneously with covariates (e.g., BMI, demographics, EMA compliance). Generalized linear models are appropriate for detecting moderate to large effects, as expected in this study, despite limits to statistical power. However, analyses were considered exploratory due to this study’s modest sample size.

EMA compliance was computed by dividing the number of completed EMA prompts by the total number of EMA prompts delivered. In order to be included in data analysis, participants had to meet a minimum EMA compliance threshold defined as completing ≥40% of EMA prompts. This criterion was met by 100% of participants. Setting a minimum threshold for EMA compliance is standard in EMA research to ensure data validity [42] and the threshold of ≥40% compliance was drawn from prior work [43].

## 3. Results

First we examined the descriptive and demographic data of the sample (see Table 2). Age ranged from 18–60 years old (*M* = 25 ± 9). Participants identified as bisexual or pansexual (62%), lesbian or homosexual (33%), and queer (5%). Participants identified as cisgender (96%) and gender-queer/gender non-conforming (4%). The sample BMI ranged from 25 to 45 (*M* = 32 ± 5). The distribution of weight categories included overweight (36%), obese (29%), and very obese (35%). The sample was racially diverse (e.g., 45% identified as a member of a racial or ethnic minority group or as multiracial) and highly educated (e.g., most completed at least some college). All women who completed the baseline visit also completed EMA procedures and no attrition occurred during the EMA period. Compliance to EMA procedures was excellent (*M* = 76% of prompts completed). Most participants (73%) completed at least 70% of prompts and no participants completed less than 40%.

### 3.1. Perceived Experiences of Lifetime Weight Stigma at Baseline

All sexual minority women in the sample (100%) endorsed experiencing weight stigma at least once in their lives (*M* = 1.24 ± 0.79). All categories of weight stigma were endorsed by at least some members of the sample. The most common forms of lifetime weight stigma were negative comments from others (reported by 96% of participants), family (93%), children (87%), and doctors (84%). See Table 3 for details.

### 3.2. Perceived Experiences of Momentary Weight Stigma in Daily Life

#### 3.2.1. Frequency of Perceived Momentary Weight Stigma Experiences

Across the sample, participants reported 44 instances of momentary weight stigma (*M* = 0.80 ± 2.12), with 24% of participants reporting at least one stigma event over the five-day EMA period. The frequency of weight stigma reported by individual participants ranged from 0–12 events. Of those reporting weight stigma during EMA, 54% identified as bisexual/pansexual, 46% identified as gay/lesbian, 23% had a BMI of 25–29.99 kg/m^2^, 31% had a BMI of 30–34.99 kg/m^2^, and 46% had a BMI of 35 kg/m^2^ or more.

Exploratory analyses using generalized linear models showed strong positive associations between lifetime weight stigma at baseline and weight stigma frequency reported during EMA, even when accounting for covariates like BMI, demographics, and EMA compliance (*b* = 1.0, *SE* = 0.30, *p* = 0.001, *RR* = 2.72). Weight stigma frequency during EMA was also positively related to participant BMI (*b* = 0.12, *SE* = 0.29, *p* < 0.001, *RR* = 1.13), age (*b* = 0.06, *SE* = 0.01, *p* < 0.001, *RR* = 1.07), and education level (*b* = 0.57, *SE* = 0.09, *p* < 0.001, *RR* = 1.77), but not to race or EMA compliance (*p* > 0.05).

#### 3.2.2. Types of Perceived Momentary Weight Stigma

Most weight stigma events reported by sexual minority women with overweight/obesity in daily life involved being glared at or singled out (30% of events) or encountering physical barriers (25%). See Table 4 for full details.

#### 3.2.3. Perceived Reason(s) for Momentary Stigma in Addition to Weight

When sexual minority women reported experiencing momentary stigma in daily life, they were asked to report all perceived reasons they were stigmatized using a checklist of potential reasons. During most instances of perceived weight stigma (75% of events) in daily life, sexual minority women reported being stigmatized because of their weight *and* another marginalized identity. For example, 43% of perceived weight stigma events also involved stigma due to sexual orientation, 41% also involved stigma due to age, 25% also involved stigma due to gender, and 20% also involved stigma due to race. Though reported less frequently, 5% also involved stigma due to SES. Only 25% of perceived weight stigma events reported by sexual minority women with overweight/obesity were solely attributed to weight.

#### 3.2.4. Affective Response to Perceived Momentary Weight Stigma

Most perceived weight stigma events made participants feel upset (36%) or slightly bothered (34%), and 27% of weight stigma events were extremely upsetting. Only 2% of weight stigma events did not bother participants.

#### 3.2.5. Intensity of Perceived Momentary Weight Stigma

Using a 0 (mild) to 10 (severe) scale, participants rated the average intensity of perceived weight stigma events to be 5.66 (*SD* = 2.44). Intensity ratings for individual weight stigma events ranged from 0 to 10, with most events (66%) rated as moderately to severely intense (5 or more out of 10) and only 9% rated as mild to low intensity (2 or less).

#### 3.2.6. Location of Perceived Momentary Weight Stigma

Perceived weight stigma events reported in daily life occurred at participants’ place of work (30% of events), a general public setting (27%), on public transportation (18%), at home (11%), at a gym, park, or fitness center (7%), or at a restaurant or bar (2%).

#### 3.2.7. Sources of Perceived Momentary Weight Stigma

As seen in Table 4, participants reported perceived weight stigma from varied sources, most commonly children and teens (28% of events), retail workers (23%), and strangers (21%).

#### 3.2.8. Qualitative Descriptions of Perceived Momentary Weight Stigma

See Table 5.

## 4. Discussion

Findings from this descriptive study showed that all sexual minority women of higher body weight in this sample reported experiencing weight stigma at some point in their lives, with nearly one quarter of participants also reporting weight stigma experiences during a short, five-day EMA period. The high rates of perceived weight stigma reported in this sample are comparable to weight stigma rates reported in individuals with overweight/obesity of sexual minority status *and* from the general population [20], suggesting that weight stigma is a common experience for adults of larger body size regardless of sexual orientation. This is significant because historical perspectives have proposed that sexual minority status may protect against heteronormative body ideals and associated pressure/weight stigma in women [44], yet the findings showed that none of the sexual minority women with overweight/obesity in this study had been protected from weight stigma experiences. Further, when sexual minority women in this study reported weight stigma in daily life, most events involved being stigmatized because of weight *and* other reasons, most commonly sexual orientation. This finding reveals that among sexual minority women, weight stigma may be experienced in tandem with stigma due to other marginalized identities. This suggests that assessing weight stigma in isolation may not detect the full complexity of the stigma experience. Thus, it is critical for future research to assess how weight stigma intersects with other sources of stigma to affect health among sexual minority women and other marginalized groups.

Although weight stigma is a well-established problem affecting many adults of higher body weight [2], sexual orientation is rarely assessed or examined in these studies, making sexual minority women’s weight stigma experiences imperceptible. Study data give voice to these experiences, showing that 100% of sexual minority women with overweight/obesity in this sample endorsed at least one instance of perceived lifetime weight stigma, averaging one to several events in their lives by young adulthood to midlife (*M*_SSI_ = 1.24). Even during just a five-day EMA period, 24% of women reported 44 weight stigma events (*M* = 0.16 events/participant/day). These rates of lifetime and daily perceived weight stigma mirror those seen in adults with overweight/obesity from the general population, who report average weight stigma rates of about one to several lifetime events MSSI = 0.81–1.78; [22,45,46,47,48,49] and 0.11–3.08 daily events in EMA studies [28,29,30]. The pervasiveness of lifetime weight stigma among women in this sample is comparable to rates reported among sexual minority individuals with overweight/obesity [20], though we are not aware of any EMA studies assessing weight stigma in this group. It was also notable that all 11 types of perceived weight stigma assessed on the SSI were endorsed by at least some sexual minority women in this study, including more severe forms like job discrimination and physical violence. Given the pervasiveness of perceived weight stigma found in this sample, it is critical for future research to evaluate sexual minority women’s weight stigma experiences and to assess and report sexual orientation in weight stigma research.

This point is further strengthened by EMA data revealing that during most momentary weight stigma events, sexual minority women perceived the cause of stigma to be their weight in addition to another minority identity, most commonly sexual orientation, gender, age, and race. This finding suggests that among sexual minority women, weight stigma is not an isolated issue with respect to body size. Rather, weight bias may be an added source of stigma that occurs alongside stigma based on other aspects of identity (e.g., sexual orientation). This interpretation aligns with double discrimination theory [50], which views stigma as an additive experience, such that those who face multiple sources of stigma (e.g., sexual minority women with obesity) experience higher levels of stigma than both their counterparts who are privileged (e.g., heterosexual men) *and* those with only one source of social disadvantage (e.g., heterosexual women; [51,52]). However, it is also possible that the multiple social identities held by sexual minority women intersect to produce a unique qualitative experience of weight stigma in this group, as suggested by the frequent convergence of stigma due to weight and sexual orientation in this sample. This is the implication of intersectionality theory [53,54,55], which proposes that multiple social identities operate synergistically (rather than additively) to influence social position, stigma, and health. Although data from this uncontrolled pilot study cannot directly test the applicability of these theories, one key implication for future research is that assessing weight stigma in isolation may not sufficiently capture the complexity of sexual minority women’s stigma experiences. Thus, it is critical to assess sexual orientation in weight stigma research and to assess weight stigma in the context of other marginalized identities. Double discrimination [50] and intersectionality theories [55] may be useful frameworks for advancing this research agenda.

The finding that sexual minority women experienced weight stigma in tandem with other stigma sources merits further study because it may also have vital implications for health. Prior work has shown that experiencing multiple simultaneous sources of stigma has compounding negative health effects [51,52,54,56]. The emphasis here is on individuals’ subjective perceptions of stigma because multiple marginalization often manifests in ambiguous ways that are difficult to “objectively” verify, and the perception of stress plays a key role in driving downstream negative health consequences [57,58]. Indeed, complex factors may affect how different sources of stigma interact to impact health: weight, sexual orientation, and other aspects of identity vary considerably in their prevalence, concealability, perceived controllability, and associated group pride, and related attitudes and norms differ across cultural groups and geographic regions [4,59,60]. This complexity underscores the need for research examining the effects of intersecting weight stigma on sexual minority women’s health. To promote resilience, it may also be vital to identify protective factors (e.g., social support, identification with a community identity) that may buffer negative health consequences [61,62].

The study findings also have implications for treatment. Most perceived weight stigma events reported by sexual minority women in this sample occurred alongside other sources of stigma, yet few stigma- or weight-related interventions address multiple minority identities. This may position multiple-marginalized groups like sexual minority women to receive treatments that may be less relevant and efficacious in meeting their needs. Research is needed to test the benefits of tailoring treatments to address multiple sources of stigma for sexual minority women or to expand stigma reduction interventions to address multiple stigma sources.

Data from this study also have broader treatment implications. Although we leveraged EMA in this study to assess perceived weight stigma experiences, ecologically valid methods can also be leveraged to deliver in-the-moment interventions to support sexual minority women in the moments that they experience stigma to reduce stress and possibly mitigate effects on health [63]. The pervasiveness of perceived weight stigma in this sample and existing research showing a high prevalence of weight stigma in the general population points to the need for systemic interventions to reduce the perpetration of stigma [64]. Given the findings that weight stigma was common in the workplace and from children/teens, systemic efforts may involve delivering workplace interventions targeting multi-level change or delivering anti-bullying programs to children/teens to disrupt early internalization of negative stereotypes [65]

Another novel finding from this study was that for a subset of participants, perceived weight stigma events may occur more frequently in daily life than indicated on one-time retrospective assessments. Similar findings are seen in EMA studies of weight stigma in adults with overweight/obesity from the general population [29,30]. Participants in this study reported ‘one to several weight stigma events per lifetime’ at baseline, yet 24% of participants reported 44 weight stigma events over a short five-day period. Providing additional context, one study showed that 26% of lesbian women reported weight stigma events in the past month using a one-time assessment [21], and we showed a similar rate over just five days. These findings suggest that EMA is a vital assessment strategy for understanding the frequency and characteristics of individuals’ day-to-day perceived weight stigma experiences.

Finally, aside from compounding reasons for stigma, most data on the characteristics and context of weight stigma events in daily life revealed more similarities than differences between sexual minority women and other adults with overweight/obesity. Women in this sample reported that most daily perceived weight stigma events involved verbal comments from others and physical barriers in the environment, with the most common sources being strangers (e.g., children/teens, retail workers), friends/family, and environmental barriers. Stigma events most often occurred in the places where people spend the most time (e.g., at work, in public, at home), and nearly all stigma events were emotionally upsetting. These findings share many similarities with reports from EMA studies assessing perceived weight stigma among adults with obesity, suggesting that some aspects of the weight stigma experience may be universal [28,29,30,66]. However, given this study’s limited sample size and duration of EMA, future EMA studies using larger samples are needed to directly test these claims.

### Limitations

The strengths of this study include its novelty, as this study is among the first to assess sexual minority women’s experiences of lifetime and daily perceived weight stigma using the SSI measure and EMA methods. Participants in this study also demonstrated excellent compliance to EMA procedures, strengthening the validity of our EMA findings.

However, this study also has key limitations. This study was designed to be a relatively small, innovative project to provide pilot data for future, larger studies. Thus, our assessment of naturalistic weight stigma experiences was limited to a five-day period, a brief window that may underestimate how many sexual minority women in this sample experience weight stigma in daily life. This study also recruited a convenience sample of modest size, meaning that study data cannot be generalized to shed light on the prevalence of weight stigma experiences in the broader population of sexual minority women with overweight/obesity. Further, 24% of participants reported 44 perceived weight stigma events during the EMA period. Thus, study data on the characteristics of momentary weight stigma are based on small cell sizes, and specific findings regarding stigma form and context should not be over-generalized. Rather, these data should be seen as a starting point to inform future studies with larger, more representative samples.

This study also had methodological limitations. Due to resource limitations, this study did not include a control group of heterosexual women with overweight/obesity. Thus, we cannot compare the perceived weight stigma experiences of sexual minority women in this sample to their heterosexual counterparts, preventing direct tests of intersectionality or double discrimination theory. Future controlled studies that are guided by these frameworks are needed to understand how sexual minority status may influence perceived weight stigma experiences among women.

Several methodological factors may limit the interpretations of EMA research. The repeated administration of EMA measures can contribute to participant burden, missing data, and potential reactivity effects, though these limitations are debated within the field [25]. To minimize these effects, study prompts were designed to be brief and their order was randomized. Additionally, there are no widely validated EMA measures of momentary stigma, and thus this is an important area for further development.

Several factors may limit the generalizability of study findings. This study recruited sexual minority women from a Northeast region of the United States, and thus findings may not generalize to other geographical areas and countries where cultural attitudes related to sexuality, gender, and weight may differ. This study also examined perceived weight stigma experiences in a sample of lesbian, bisexual, and queer women together. A strength of this approach is that our data reflect the experiences of varied sexual minority women, but we are limited by insufficient statistical power to examine how individual subgroups of sexual minority women may differentially experience weight stigma. This is an important direction for future work.

Although these larger studies are critically needed to investigate weight stigma experiences in this population, given the dearth of prior research we believe the data from this study provide an important starting point to inform the design and implementation of larger EMA studies investigating weight stigma among sexual minority women.

## 5. Conclusions

Findings showed high rates of perceived lifetime weight stigma among sexual minority women with overweight/obesity in this sample and frequent co-occurrence of perceived weight stigma with stigma due to other marginalized identities (e.g., sexual orientation) in daily life. Given recent evidence that weight stigma negatively affects physical health in addition to quality of life, future research must aid in the reduction of weight stigma and its impact on health in this group.

## Figures and Tables

**Table 1:** Items used to assess momentary stigma events in daily life.

Construct Assessed	Assessment Question Used	Response Options
**Stigma occurrence and type**	Since the last prompt, have you experienced stigma (i.e., felt you were perceived differently, treated differently, or singled out by others or the environment because of your sexual orientation, weight, gender, or another part of your identity?). Check all stigmatizing situations that have occurred since the last prompt.	Multi-select checklist included: I have not experienced stigma; I was made fun of, teased, or called derogatory names; I was treated unfairly or prevented from doing something; I was made to feel inferior; I was excluded; I was glared at or singled out; I was judged or criticized; Others made unfair assumptions about me; I was treated with less courtesy/respect than others; I felt singled out by the environment; I felt family/friends were ashamed of me; I was harassed, threatened or followed; Unrelated problems were blamed on my sexual orientation, gender, weight, etc.; I overheard disparaging comments about my group; Other; Unsure.
**Stigma frequency**	How many separate times did you experience stigma since the last prompt?	Number wheel.
**Perceived reason(s) for stigma**	In your opinion, what was the reason you were stigmatized?	Multi-select checklist included: My weight; my sexual orientation; my gender; my race; my age; my social class; my religion; other; none
**Affective response to stigma**	How did you respond to this event?	Likert scale included: 1 = this event made me feel good; 2 = It did not bother me; 3 = It bothered me slightly; 4 = It upset me; 5 = This event upset me extremely
**Stigma intensity**	How intense was the stigmatization?	Likert scale included: 0 (mild) to 10 (severe)
**Stigma location**	Where did the stigmatizing event occur?	Multiple choice included: Work; home; healthcare setting; recreational setting; restaurant; public transportation; public setting; other
**Stigma source**	Who or what treated you unfairly?	Free-response
**Qualitative description of stigma**	Briefly, what happened that made you feel stigmatized?	Free-response

**Table 2:** Participant Characteristics.

Baseline Measures	Value
Age (years; mean ± SD)	25.0 (± 9.3)
Race (%)	
Caucasian/White	55%
Black/African-American	7%
Asian	13%
Hispanic/Latino	7%
More than one race	18%
Body Mass Index (kg/m^2^; mean ± SD)	32.5 (± 4.9)
Education (%)	
High school graduate or equivalent	7%
Bachelor’s degree/some college	67%
Associate’s degree	7%
Graduate degree/some graduate school	18%
Gender Identity (%)	
Cisgender	96%
Gender-queer/gender non-conforming	4%

*Notes:* SD, standard deviation.

**Table 3:** Lifetime weight stigma as measured by the Stigmatizing Situations Inventory.

Lifetime Weight Stigma (SSI Subscales)	% of Participants Who Experienced the Situation	Mean	SD	Range	Subscale α
People making negative assumptions about you	69%	1.68	1.72	0–6.00	0.84
Being excluded	62%	1.55	1.81	0–6.50	0.45
Comments from children	87%	1.55	1.40	0–4.75	0.70
Comments from others	96%	1.43	0.93	0–3.64	0.75
Comments from family	93%	1.42	0.99	0–3.71	0.64
Being stared at	80%	1.30	1.28	0–4.20	0.71
Comments from doctors	84%	1.22	1.12	0–4.50	0.79
Physical barriers or obstacles	83%	0.97	0.78	0–3.29	0.64
Loved ones being embarrassed by your size	60%	0.86	1.14	0–4.67	0.50
Physical violence	14%	0.49	1.56	0–7.00	n/a
Job discrimination	26%	0.28	0.62	0–3.00	0.41
Overall score	100%	1.24	0.79	0.02–2.98	n/a

Notes: SD, standard deviation; α = coefficient alpha. (0 = Never, 1 = Once in your life, 2 = Several times in your life, 3 = About once a year, 4 = Several times a year, 5 = About once a month, 6 = Several times a month, 7 = About once a week, 8 = Several times a week, 9 = Daily).

**Table 4:** Types and sources of perceived momentary weight stigma reported in daily life.

**Types of Perceived Weight Stigma**	**Momentary Weight Stigma Events**	
	***N***	**% of Events**
Glared at or singled out	13	30%
Environmental barriers	11	25%
Others made unfair assumptions	6	14%
Judged or criticized	4	9%
Made to feel inferior	3	7%
Felt that family/friends were ashamed	3	7%
Unrelated problems blamed on weight	2	5%
Excluded	1	2%
Treated with less courtesy/respect	1	2%
**Sources of Perceived Weight Stigma**	***N***	**% of Events**
Children/teens	12	28%
Retail workers (e.g., store clerks)	10	23%
Strangers	9	21%
Family	4	9%
Friends/Peers	4	9%
The environment (e.g., not being able to fit into clothing)	4	9%
Professionals (e.g., a client)	1	2%

**Table 5:** Qualitative descriptions of perceived weight stigma events in daily life.

What Happened that Made You Feel Stigmatized?
A bad situation I was in was blamed on my “unhealthy habits”, aka gaining some weight at the time.
People stared at me while at the bus stop because I was slouching. Maybe it was because I was wearing a short sleeved shirt in chilly weather because I get easily warm because of my weight.
I could not find clothes that looked good when it was going to be warm.
My friend’s child was complimenting my dress and then said that I looked like I was having a baby. I feel really confident in the dress I’m wearing and have felt that it camouflaged my belly well but I guess I’m wrong.
My son made negative comments about me.
The teachers and students sometimes assumed that I am lazy or stupid.
The students made fun of me.
Could not find my clothes size. The woman helping me kept making noises.
In the grocery store, I expressed out loud my desire to eat pizza for dinner to my mother, which prompted a stranger to turn their head and stare.
I was offered clothes that were too small.
Because of my appearance, someone assumed that I was single.
I couldn’t find clothes that fit or were flattering for my figure.
Felt that I shouldn’t go into a physical therapy pool due to my size and me wearing men’s swim shorts and a t-shirt.
When I sat in the seat on the bus, my lower body touched the people next to me because the seat was small.
The students disrespected me.
I was ignored.
Trying to fit through the tables at school.
Glaring at me and sighing.
